# Urinary Resveratrol Metabolites Output: Differential Associations with Cardiometabolic Markers and Liver Enzymes in House-Dwelling Subjects Featuring Metabolic Syndrome

**DOI:** 10.3390/molecules25184340

**Published:** 2020-09-22

**Authors:** Vanessa Bullón-Vela, Itziar Abete, Maria Angeles Zulet, Yifan Xu, Miguel A. Martínez-González, Carmen Sayón-Orea, Miguel Ruiz-Canela, Estefanía Toledo, Vicente Martín Sánchez, Ramon Estruch, Rosa María Lamuela-Raventós, Enrique Almanza-Aguilera, Montserrat Fitó, Jordi Salas-Salvadó, Andrés Díaz-López, Francisco J. Tinahones, Josep A. Tur, Dora Romaguera, Jadwiga Konieczna, Xavier Pintó, Lidia Daimiel, Ana Rodriguez-Mateos, José Alfredo Martínez

**Affiliations:** 1 Department of Nutrition, Food Science and Physiology, Center for Nutrition Research, University of Navarra, 31008 Pamplona, Spain; mbullon@alumni.unav.es (V.B.-V.); mazulet@unav.es (M.A.Z.); jalfmtz@unav.es (J.A.M.); 2 Consorcio CIBER, M.P. Fisiopatología de la Obesidad y Nutrición (CIBERObn), Instituto de Salud Carlos III (ISCIII), 28029 Madrid, Spain; mamartinez@unav.es (M.A.M.-G.); msayon@unav.es (C.S.-O.); mcanela@unav.es (M.R.-C.); ETOLEDO@unav.es (E.T.); esacane@clinic.cat (R.E.); lamuela@ub.edu (R.M.L.-R.); mfito@imim.es (M.F.); jordi.salas@urv.cat (J.S.-S.); andres.diaz@urv.cat (A.D.-L.); fjtinahones@uma.es (F.J.T.); pep.tur@uib.es (J.A.T.); doraromaguera@yahoo.es (D.R.); jadzia.konieczna@gmail.com (J.K.); xpinto@bellvitgehospital.cat (X.P.); 3 Department of Nutritional Sciences, School of Life Course Sciences, Faculty of Life Sciences and Medicine, King’s College London, London SE1 9NH, UK; yifan.xu@kcl.ac.uk (Y.X.); ana.rodriguez-mateos@kcl.ac.uk (A.R.-M.); 4 Department of Preventive Medicine and Public Health, University of Navarra, 31008 Pamplona, Spain; 5 Centro de Investigación Biomédica en Red de Epidemiología y Salud Pública (CIBERESP), Instituto de Salud Carlos III (ISCIII), 28029 Madrid, Spain; vicente.martin@unileon.es; 6 Institute of Biomedicine (IBIOMED), University of León, 24071 León, Spain; 7 Department of Internal Medicine, IDIBAPS, Hospital Clinic, University of Barcelona, 08036 Barcelona, Spain; 8 Department of Nutrition, Food Sciences and Gastronomy, XaRTA, INSA-UB, School of Pharmacy and Food Sciences, Nutrition and Food Safety Research Institute, University of Barcelona, 08028 Barcelona, Spain; 9 Cardiovascular Risk and Nutrition Research Group (CARIN), Hospital del Mar Research Institute (IMIM), 08007 Barcelona, Spain; ealmanzaa@outlook.com; 10 CIBER Fragilidad y Envejecimiento Saludable (CIBERFES), Institute of Health Carlos III, 28029 Madrid, Spain; 11 Institute of Nutrition and Food Safety (INSA-UB), University of Barcelona, Santa Coloma de Gramenet, 08921 Barcelona, Spain; 12 Departament de Bioquímica i Biotecnologia, Universitat Rovira i Virgili, Unitat de Nutrició Humana, 43201 Reus, Spain; 13 Institut d’Investigació Pere Virgili (IISPV), Hospital Universitari Sant Joan de Reus, 43204 Reus, Spain; 14 Department of Endocrinology, Instituto de Investigación Biomédica de Málaga-IBIMA, University of Málaga, Virgen de la Victoria Hospital, 29010 Málaga, Spain; 15 Research Group on Community Nutrition & Oxidative Stress, University of Balearic Islands, 07122 Palma de Mallorca, Spain; 16 Research Group on Nutritional Epidemiology & Cardiovascular Physiopathology (NUTRECOR), Health Research Institute of the Balearic Islands (IdISBa), University Hospital Son Espases (HUSE), 07120 Palma de Mallorca, Spain; 17 Lipids and Vascular Risk Unit, Internal Medicine, Hospital Universitario de Bellvitge, Hospitalet de Llobregat, 08908 Barcelona, Spain; 18 Precision Nutrition Program, IMDEA Food, CEI UAM + CSIC, 28049 Madrid, Spain; lidia.daimiel@imdea.org

**Keywords:** antioxidant, inflammation, liver enzymes, metabolic syndrome, non-alcoholic fatty liver disease, resveratrol

## Abstract

Metabolic syndrome (MetS) components are strongly associated with increased risk of non-alcoholic fatty liver disease (NAFLD) development. Several studies have supported that resveratrol is associated with anti-inflammatory and antioxidant effects on health status. The main objective of this study was to assess the putative associations between some urinary resveratrol phase II metabolites, cardiometabolic, and liver markers in individuals diagnosed with MetS. In this cross-sectional study, 266 participants from PREDIMED Plus study (PREvención con DIeta MEDiterránea) were divided into tertiles of total urinary resveratrol phase II metabolites (sum of five resveratrol conjugation metabolites). Urinary resveratrol metabolites were analyzed by ultra- performance liquid chromatography coupled to triple quadrupole mass spectrometry (UPLC-Q-q-Q MS), followed by micro-solid phase extraction (µ-SPE) method. Liver function markers were assessed using serum levels of aspartate aminotransferase (AST), alanine aminotransferase (ALT), and gamma-glutamyl transferase (GGT). Moreover, lipid profile was measured by triglycerides, very-low-density lipoprotein cholesterol (VLDL-c), and total cholesterol/high-density lipoprotein ratio (total cholesterol/HDL). Linear regression adjusted models showed that participants with higher total urine resveratrol concentrations exhibited improved lipid and liver markers compared to the lowest tertile. For lipid determinations: log triglycerides (β_T3_ *=* −0.15, 95% CI; −0.28, −0.02, *p*-trend = 0.030), VLDL-c, (β_T3_ *=* −4.21, 95% CI; −7.97, −0.46, *p*-trend = 0.039), total cholesterol/HDL ratio Moreover, (β_T3_ *=* −0.35, 95% CI; −0.66, −0.03, *p*-trend = 0.241). For liver enzymes: log AST (β_T3_ *=* −0.12, 95% CI; −0.22, −0.02, *p*-trend = 0.011, and log GGT (β_T3_ *=* −0.24, 95% CI; −0.42, −0.06, *p*-trend = 0.002). However, there is no difference found on glucose variables between groups. To investigate the risk of elevated serum liver markers, flexible regression models indicated that total urine resveratrol metabolites were associated with a lower risk of higher ALT (169.2 to 1314.3 nmol/g creatinine), AST (599.9 to 893.8 nmol/g creatinine), and GGT levels (169.2 to 893.8 nmol/g creatinine). These results suggested that higher urinary concentrations of some resveratrol metabolites might be associated with better lipid profile and hepatic serum enzymes. Moreover, urinary resveratrol excreted showed a reduced odds ratio for higher liver enzymes, which are linked to NAFLD.

## 1. Introduction

Metabolic syndrome (MetS) encompasses several clinical conditions, including central obesity, hypertension, dyslipidemia, and insulin resistance leading to an inflammatory state [1], which is frequently accompanied by liver dysfunction [2]. Many clinical studies suggested that non-alcoholic fatty liver disease (NAFLD) is the liver manifestation of MetS [2,3,4]. NAFLD is characterized by simple hepatic steatosis (excessive triglyceride accumulation) leading to alterations in oxidative and inflammatory pathways. This state promotes non-alcoholic steatohepatitis (NASH), subsequently cirrhosis and hepatic carcinoma in last stages [3,5]. The prevalence of NAFLD increases with rates of obesity and type 2 diabetes mellitus (T2DM), mainly due to unhealthy lifestyle behaviors [2]. It has been suggested that insulin resistance and abnormal lipid profile were strongly involved in NAFLD pathogenesis and prognosis [3]. Hyperinsulinemia increases free fatty acid levels promoting a disrupted flux of triglycerides into hepatocytes [5,6,7]. In NAFLD, commonly abnormal elevation of serum levels of alanine aminotransferase (ALT), aspartate aminotransferase (AST), and gamma-glutamyl transferase (GGT) have presented [3,4]. Liver biopsy is still the gold standard for diagnosing NAFLD, but some limitations regarding high cost and invasive nature hindered it being applicable in epidemiological studies [3]. In this sense, non-invasive liver markers such as transaminases, fatty liver index (FLI) and hepatic steatosis index (HSI) are recommended in individuals with obesity and MetS as a routine work-up to identify the risk of NAFLD and subjects with worse prognosis [4]. Metabolomics is the technology that analyses metabolites in a biological system, and it has been considered as a potential omics tool to investigate the impact of nutrients, foods, and dietary patterns on human health with application in precision nutrition research [8]. Moreover, metabolite biomarkers related to dietary intake could be useful as potential non-invasive biomarkers of effects and disease risk [8].

Lifestyle interventions focused on weight loss, exercise, and a healthy diet can improve the histopathological and clinical features of NAFLD [3]. Scientific evidence suggests that the modulation of dietary components can influence NAFLD pathogenesis beyond caloric restriction [9]. In this sense, many epidemiological and clinical data support that the beneficial effects of the Mediterranean diet (MedDiet) on metabolic disturbances linked to NAFLD mainly attributed to higher consumption of bioactive compounds, such as resveratrol and anthocyanins that are present in whole-grain cereals, fruits, vegetables, healthy fatty acids, and moderate intake of wine [10,11,12,13,14,15]. Resveratrol is a member of the stilbene family that is present in several foods and plants [16,17]. The primary dietary sources are red grapes and red wine, with smaller amounts present in peanuts, berries, and dark chocolate [16,18]. After the intake, resveratrol enters the gastrointestinal tract and then the liver via the hepatic portal system, and is metabolized by phase II enzymes generating sulfate (*trans*-/*cis*- resveratrol 3-*O*-sulfate and 4-*O*-sulfate) and glucuronide (*trans*-/*cis*-resveratrol 3-*O*-glucuronide and 4-*O*-glucuronide) metabolites [19,20]. The gut microbiota can metabolize the resveratrol and conjugated metabolites into dihydro-resveratrol and lunularin [19]. Several human studies have shown that the most abundant resveratrol phase II conjugates are glucuronides and sulfate metabolites in urine and plasma samples [20,21]. Limited information has been reported regarding bioavailability and pharmacokinetics of glycosylated metabolites (piceid), derived from gut microbiota and other stilbenes (piceatannol). Pharmacokinetics studies in human showed that resveratrol is highly metabolized, but it has low bioavailability [20,22,23]. In a study used radiolabeled ^14^C-resveratrol to evaluate the bioavailability of resveratrol intake in humans, results indicated that 70% of the resveratrol absorption was recovered in urine. Moreover, the rapid sulfate conjugation by the intestine-liver could be the principal influence of their bioavailability [22]. Moreover, the bioavailability and quantity of resveratrol metabolites can be affected by several factors leading to a significant interindividual variability [19,24,25]. Despite its low bioavailability, several studies have reported that resveratrol metabolites exert beneficial effects modulating inflammatory and oxidative pathways related to several chronic diseases, such as cancer, cardiovascular disease (CVD), T2DM, obesity, and NAFLD [21]. NAFLD, most of the studies on resveratrol, and the mechanism of action have been developed in vitro and animal rodents, which used higher resveratrol concentrations, different cell lines, and animal models that often overlap [26]. The protective effects of resveratrol on NAFLD mainly include improvements in principal risk factors, such as blood glucose and insulin levels [27], lipid metabolism [28], and liver damage [29], but results have remained inconclusive. In this regard, knowledge of the underlying effects of resveratrol metabolites on liver markers and risk of NAFLD in individuals diagnosed with MetS is still needed. Thus, our objective was to determinate the potential association between some phase II urinary metabolites of resveratrol and cardiometabolic and liver markers in individuals diagnosed with Mets. We hypothesized that high urinary resveratrol metabolites would be associated with a favorable cardiometabolic profile and hepatic markers related to the risk of NAFLD development.

## 2. Results

### 2.1. Participant Characteristics

Baseline sociodemographic, clinical, and liver characteristics stratified by sex are summarized in Table 1. This study included subjects between 55 to 75 years old (65.8 ± 5.1 years) who were overweight or obese (32.2 ± 3.4 kg/m^2^). Men were more prevalent as former smokers, had higher waist circumference (109.9 ± 8.5 cm), and visceral fat mass (2850.3 ± 826.2 g) compared to women (all *p*-values < 0.05). Moreover, men showed higher levels of physical activity (3690 ± 3101.4 Metabolic Equivalent of Task (MET)/min/week) (*p* > 0.001). There were no differences in taking lipid-lowering and anti-diabetic medications in between genders. Likewise, glucose, homeostatic model assessment for insulin (HOMA-IR), triglycerides, and very-low-density lipoprotein cholesterol (VLDL-c) did not differ among sexes. Nevertheless, women had higher insulin, cholesterol, high-density lipoprotein cholesterol (HDL-c), low-density lipoprotein cholesterol (LDL-c) levels in comparison to men (all *p* > 0.05). Concerning liver markers, ALT (31.2 ± 24.5 U/L), AST (25.2 ± 16.3 U/L), and FLI (80.4 ± 14.4) were significantly higher in men than women (all *p*-values > 0.05). Moreover, there was significant difference in the percentage of participants with ALT values above the upper limit normal (ULN) between men and women. Meanwhile, women had higher HSI (44.0 ± 5.1) in comparison to men (*p* = 0.004). Regarding dietary intake and urine resveratrol metabolites (Table 2), the intake of macronutrients did not differ significantly between genders. However, men had higher energy (2689.4 ± 534.5 kcal/d, *p* = 0.004) intake and polyunsaturated fatty acid (PUFA) consumption (19.1 ± 7.2 g/d, *p* = 0.049) compared to women. Nevertheless, women consumed more vegetables (353.0 ± 124.0 g/d, *p* = 0.024) and had a lower grape intake (5.8 ± 12.3 g/d, *p* = 0.012). Moreover, men had a much higher alcohol consumption than women (19.7 ± 19.8 g/d, *p* < 0.001) with statistically significant differences in total red, young red, aged red, and rose wine consumption between sexes.

Urine resveratrol metabolites (Table 2) showed that men had higher *trans*-resveratrol-3-*O*-sulfate and *cis*-resveratrol-3-*O*-glucuronide/*cis*-resveratrol-4′-*O*-glucuronide urine levels (all *p*-values < 0.05) compared to women.

### 2.2. Association between Total Urine Resveratrol Metabolites Concentrations, Cardiometabolic Profile, and Liver Markers

The association between total urine resveratrol metabolites and glucose metabolism markers, blood lipid, and liver markers are shown in Table 3, Table 4 and Table 5, respectively. After adjusting for covariates, there were no significant associations between total urine resveratrol metabolites and glucose metabolism markers (Table 3). Table 4 summarizes values concerning lipid metabolism. No significant associations were observed between total urine resveratrol metabolites and LDL-c and log triglyceride/HDL ratio among tertiles. Although in the adjusted model, participants in the T3 had significantly lower levels of total cholesterol compared to T1 (β_T3_ = 11.67, 95% CI, −22.21 to −1.13), but there was no significant tendency among tertiles (*p*-trend = 0.108). Interestingly, individuals in the highest tertile (T3) of total urine resveratrol metabolites had significantly lower levels of log triglycerides (β_T3_ = −0.15, 95% CI, −0.28 to −0.02), VLDL-c (β_T3_ = −4.21, 95% CI, −7.97 to −0.46), and total cholesterol/HDL ratio (β_T3_ = −0.35, 95%CI, −0.66 to −0.03) after adjustment. Regarding to liver markers (Table 5), compared with those in the first tertile, individuals in the third tertile had 2.4 significant unit decrease in the log GGT (95% CI, −0.42 to −0.06; *p*-trend = 0.002) and lower log AST (β_T3_ = −0.12, 95% CI, −0.22 to −0.02; *p*-trend = 0.011). Nevertheless, the differences of other liver markers (log ALT, HSI and FLI) between levels of urinary resveratrol metabolites were not statistically significant.

### 2.3. Risk of Higher Liver Enzymes and Total Urine Resveratrol Metabolites

**Figure 1:**
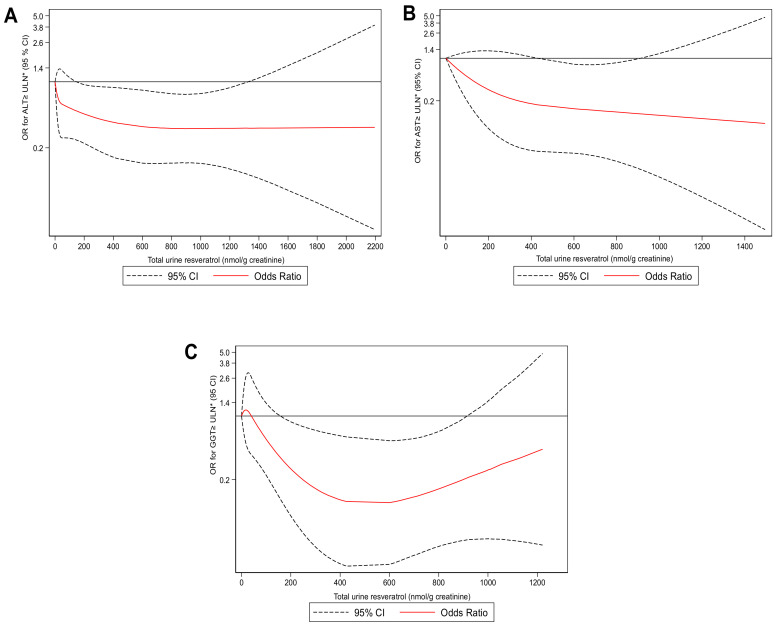
The odds ratio for liver enzymes levels above the upper limit of normal (ULN) for total urine resveratrol concentration in nmol/g creatinine. ULN range for ALT (men ≥ 30 UI/L, women ≥ 19 UI/L) (**A**), AST (men ≥ 37 UI/L, women ≥ 31 UI/L) (**B**), and GGT (men ≥ 60 UI/L, women ≥ 40 UI/L) (**C**). The smooth line represents the estimation of higher ALT, AST, and GGT levels when using zero as the reference value for total urine resveratrol metabolite (4 knots for ALT and GGT; 3 knots for AST) whereas the dashed lines indicate 95% CIs.

We tested the associations of total urine resveratrol metabolites and the risk of higher ALT (A), AST (B), and GGT (C) levels (Figure 1). Cubic splines analyses indicated that participants who had total urinary resveratrol concentration threshold had a lower odds ratio for liver enzymes above the ULN. Urinary resveratrol metabolites concentration threshold: ALT (169.2 to 1314.3 nmol/g creatinine), AST (559.9 to 893.8 nmol/g creatinine), and GGT (169.2 to 893.8 nmol/g creatinine).

## 3. Discussion

In this research, higher urine concentrations of some resveratrol phase II metabolites (total sum of *trans*-resveratrol-3-*O*-glucuronide, *trans*-resveratrol-4′-*O*-glucuronide, *cis*-resveratrol-3-*O*-glucuronide, *cis*-resveratrol-4′-*O*-glucuronide, and *trans*-resveratrol-3-*O*-sulfate) have associated with favorable lipid and liver markers in individuals diagnosed with MetS. Indeed, cubic spline models suggest that total urinary resveratrol excretion was associated with a lower risk of higher levels of liver enzymes related with increased risk of NAFLD (concentration threshold for ALT = 169.2 to 1314.3 nmol/g creatinine, AST = 559.9 to 893.8 nmol/g creatinine and GGT = 169.2 to 893.8 nmol/g creatinine), even after adjustment for potential factors. MetS components increase the risk of NAFLD development [2,3,4]. In general, our population had an abnormal metabolic profile as characteristic of MetS where women showed higher cholesterol and LDL-c levels compared to men. Meanwhile, men exhibited higher levels of ALT, AST, and FLI. Epidemiological studies evidenced that age and sex affect NAFLD prevalence [3]. Ageing involves changes in sex hormones levels, fat redistribution that increases the risk of CVD and NAFLD, especially in post-menopausal women [30,31]. Healthy dietary patterns, such MedDiet, include foods that not only might improve weight modulation, but also have several bioactive compounds like (poly)phenols with anti-inflammatory and antioxidant properties, which show beneficial metabolic effects [9,10,32,33]. Some molecules, such as anthocyanidins and resveratrol, might involve in the metabolic process involved in NAFLD [15,29]. Scientific evidence suggested that resveratrol is a multi-targeted compound for chronic diseases [21]. However, variations in the study design, small samples sizes, diverse analytical methods, and other factors trigger heterogeneous conclusions. Consequently, results should be interpreted cautiously [24,29,33,34]. Interestingly, our findings showed that individuals in the highest tertile of total urinary resveratrol metabolites had lower levels of triglycerides, VLDL-c and total cholesterol/HDL ratio compared with those in the lowest tertile. Previously, the PREDIMED study (PREvención con DIeta MEDiterránea) evaluated the association of cardiovascular risk factors and total urinary resveratrol metabolites [35]. Authors demonstrated that increased urinary resveratrol excretions were associated with higher HDL-c, lower triglycerides concentrations and decreased heart rate, but did not found associations with blood pressure [35]. While in our results, no differences were found in the HDL-c concentrations. The discrepancies between our results related to the lipid metabolism could be partly explained for the T2DM status attributable to the synergetic effects of anti-diabetic drug and resveratrol as well as lipid-lowering medication [28]. It is important to mention that incidence of T2DM in our population was lower compared to Zamora et al., reported [35]. Moreover, when we adjusted the regression models considering lipid-powering and anti-diabetic treatment, our results did not change (data not shown). Another difference between both studies is that we quantified slightly different resveratrol metabolites. We quantified *trans*-resveratrol-3-*O*-glucuronide, *trans*-resveratrol-4′-*O*-glucuronide, *cis*-resveratrol-3-*O*-glucuronide, *cis*-resveratrol-4′-*O*-glucuronide and *trans*-resveratrol-3-*O*-sulfate while Zamora-Ros et al. quantified (*trans*-/*cis*-resveratrol-3-*O*-glucuronide, *cis*-resveratrol-4′-*O*-glucuronide, *trans*-/*cis* resveratrol-4′-*O*-sulfate, *trans*-/*cis* resveratrol-3-*O*-sulfate). Furthermore, we used authentic glucuronide and sulfated standards to quantify each metabolite. In contrast, Zamora-Ros et al. used the resveratrol aglycone to quantify all glucuronide and sulfated metabolites, which can lead to errors in the quantification of glucuronide and sulfated metabolites [36]. The lack of commercially available glucuronide and sulfate resveratrol standards is still an issue that hampers advancements in the quantification of total resveratrol metabolite. The lipophilic nature of resveratrol could facilitate the entry into the surface of albumin and lipoprotein, and these properties could confer benefits on lipid profile, avoiding the oxidation of LDL [28,37,38]. A study found that resveratrol metabolites, including *trans*-/*cis*-resveratrol-3-*O*-glucuronide, and *cis*-resveratrol-3-*O*-glucoside, as well as free *trans*-resveratrol, were incorporated into the LDL of human participants after intake of moderate red wine, which could suggest the cardioprotective role of resveratrol on atherogenic markers and oxidative stress [37]. In line with our findings, a meta-analysis indicated that more prolonged resveratrol supplementation (≥6 months) with doses ranged from 8.1 to 3000 mg/d might improve triglyceride levels in subjects with T2DM [28]. However, in a prospective cohort study, Semba et al. did not find significant differences in lipid profile and inflammatory cytokines across groups of total urinary resveratrol [39]. It is essential to highlight that our participants had MetS, which are closely related to NAFLD due to deregulation of the de novo lipogenesis (DNL), insulin resistance, and hepatic triglyceride accumulation [5,6]. Thus, these findings suggested that resveratrol could improve liver parameters. Several mechanisms of resveratrol action on lipid metabolism include the activation of AMP-activated kinase (AMPK), which inhibit sterol regulatory element-binding protein 1 (SREBP-1) activity, which plays a crucial role in the DNL [28,40]. Moreover, the regulation of hepatic enzyme 3-hydroxy-3-methylglutaryl Coenzyme A (HMG-CoA) related to cholesterol synthesis [38], and the overexpression of the paraoxonase 1 (PON 1) that it has shown cardioprotective effects [41]. In fact, disrupted SREBP-1 levels, increased HMG-CoA expression and decreased PON 1 activity have evidenced in NAFLD promoting a dysregulation of lipid metabolism [28,38,40,42]. This environment stimulates the accumulation of lipids into hepatocytes (liver steatosis) increasing risk to develop NAFLD [7,43]. In the current study, there were no statistically significant differences in glucose metabolism markers among tertiles of total urine resveratrol metabolite, but so far the effect of resveratrol on glucose metabolism is unclear [44]. A randomized controlled trial did not show significant effects in HOMA-IR and fasting glucose levels after four weeks of supplementation with 150 mg of *trans*-resveratrol in subjects who were overweight [45]. In contrast, a recent meta-analysis has shown that resveratrol supplementation (≥100 mg/d) might reduce levels of insulin and glucose in individuals diagnosed with T2DM [27]. Regarding liver parameters, 46% of the participants had ALT values above ULN, and higher total urinary resveratrol metabolites were significantly associated with lower AST and GGT levels. We also observed in the cubic spline analyses that total urinary resveratrol metabolite (concentration threshold) reduced the probability of having higher liver transaminases (ALT, AST and GGT). In this respect, clinical trials studies focus on resveratrol effects on NAFLD individuals are scarce with ambiguous results [46,47]. For instance, Chen et al. showed that resveratrol supplementation (600 mg for 3 months) could decrease levels of liver transaminases, LDL-c, total cholesterol and HOMA-IR in, but did not found a significant reduction in liver steatosis. However, another study indicated that lifestyle changes focused on a healthy diet and physical activity in addition to 500 mg/d (12 weeks) of resveratrol supplementation only had beneficial effects on improvements in ALT levels and hepatic steatosis [48]. In contrast, a study showed that insulin resistance markers and hepatic steatosis remained unchanged after resveratrol supplementation [49]. Contrary to our study, Cachay et al. only included men in their study design. In fact, it has suggested that stilbene glucuronidation is more efficient in women compared to men [50]. Moreover, there are significant differences in the doses. Cachay et al. used up to 20 times higher the amount compared to other research groups. In this sense, our contrasting results can be explained by the fact that chronic higher resveratrol doses might promote saturation in absorption sites [51]. Our findings suggested that inter-individual heterogeneities might play a key role in the effectiveness of resveratrol metabolites in individuals with MetS who are overweight or obese. However, it should be noted that the studies, as mentioned above, are different in study design, populations, and several other aspects that could potentially affect results and the interpretation of their conclusions. 

There is a lack of epidemiological research in evaluating the effects of resveratrol from dietary consumption and health outcomes [21,27,28,39]. On the other hand, the majority of clinical trials assessed the effects of resveratrol supplementation using diverse resveratrol dosage and frequency of intake and heterogeneous treatment lengths. Therefore, it is difficult to interpret results and establish an effective dose and treatment, especially for the use of higher amounts, which is not applicable in a normal dietary context. Resveratrol is mainly found in wine, grapes, and grape juice [18,52,53]. In our data, wine consumption was correlated with urinary resveratrol metabolites (data not shown). However, resveratrol content can vary in the same type of fruit, climate, and grape variety for wine [53,54]. The bioavailability of resveratrol is poor, resulting from low water solubility (<0.05 mg/) that can vary according to the matrix (wine, grapes, supplements, others) [20,24,54]. Rotches-Ribalta et al. evaluated resveratrol metabolites profiles after a moderate intake of red wine and grape extract tablets in healthy men [20]. Investigators found differences in the quantification of some resveratrol metabolites due to the different resveratrol composition of both sources [20]. A large number of human and animal studies suggested that bioactive phytochemicals have therapeutic effects on chronic diseases, but several factors may affect their biological response [19,25]. The main determinants of inter-individual variation could be attributed to the gut microbiota, sex, age, lifestyle, genetics, and others [25]. In this line, it seems essential to consider that resveratrol metabolites could have beneficial effects on specific population groups, where inter-individual variances in their metabolism could confer to these discrepancies [21,25,27,28,39]. Consequently, conflicting views about the effect on the metabolic profile of resveratrol in a supplementation or food form are still unclear.

Current recommendations for NAFLD prevention, treatment, and follow-up encourage lifestyle modifications to focus on habitual physical activity and healthy dietary patterns. From a dietary point of view, it is a challenge to promote healthy diets focused on foods with high content in bioactive compounds. The MedDiet could be a preferable option to be considered since its dietary components are rich in antioxidants, which are pivotal factors for the prevention and management of NAFLD [9,10,11,12,13,14,15]. In this context, well-design epidemiological and clinical trial studies to investigate the effects of dietary resveratrol on health outcomes are crucial.

The strength of this study is the large sample size of patients with detailed clinical and biochemical data. Moreover, the use of metabolomics has been considered a reliable and innovative technique for food science and precision nutrition studies [8]. In the present study, we performed an analytical method to accurately identify and quantify resveratrol metabolites using authentic standards. However, our study has some limitations. First, for total urine analyses, not all phase II metabolites were included, and we did not use glucosides and gut microbial metabolites, which can lead to underestimation of total resveratrol metabolite levels and, therefore, influence our conclusions. Hence, data are not representative of total resveratrol intake, and we included the main resveratrol phase II metabolites reported in human studies [20,21,53] and also considering the limited availability of authentic standards. Second, liver biopsy was not performed for diagnosing NAFLD participants. In this sense, non-invasive markers were acceptable to identify patients with metabolic features at risk of developing NAFLD [4,55] Finally, this study has a cross-sectional design, and the findings cannot infer causality. Likewise, results cannot be generalized to other ethnic or age groups, because the participants were elderly diagnosed with MetS at high CVD risk. However, type I and II errors cannot be discarded, despite that, the results are plausible and with clinical relevance.

## 4. Materials and Methods

### 4.1. Study Population

Participants were volunteers from the PREDIMED-Plus study, a parallel-group multi-center randomized trial (https://www.predimedplus.com/). Details of the study design have been previously described [56]. In brief, PREDIMED-Plus study was designed to investigate the effects of an energy-reduced Mediterranean diet and a weight-loss intervention by the promotion of physical activity and behavioral support on cardiovascular endpoints [57]. Individuals were men and women (65 to 75 years) who were overweight or obese and met at least three components of the MetS [58]. The study excluded participants with excessive alcohol consumption or addiction, several medical conditions (active cancer or history of malignancy, history of previous CVD, cirrhosis or liver injury, cytotoxic agents, therapy with immunosuppressive drugs, or treatment with systemic corticosteroids [56]. All participants gave their informed consent to participate in the study. This clinical trial was conducted following the Declaration of Helsinki, and the protocol was approved by institutional ethics committees of all participant centers (http://www.isrctn.com/ISRCTN89898870). This study is a cross-sectional study using baseline database from the Navarra-Nutrition node. A total of 266 participants with feasible data in the form of spot urine specimen were included in the present study. 

### 4.2. Sociodemographic, Clinical, Anthropometric, and Body Composition Variables

Sociodemographic characteristics, lifestyle data, and medical history were collected during the baseline interview according to the study protocol [56]. Smoking habit was classified into never, former, and current smoker. Diabetes was defined according to the criteria of the American Diabetes Association guidelines [59]. Anthropometric variables were measured by trained dietitians using standardized procedures and calibrated equipment [56]. Height (in centimeters) and weight (in kilograms) were measured to calculate body mass index (BMI) (kg/m^2^). Visceral fat mass was estimated using the dual-energy X-ray absorptiometry (Lunar iDXA™, software version 6.0, Madison, WI, USA) performed by trained study staff. We used a validated Registre Gironi del Cor (REGICOR) questionnaire to assess physical activity (Metabolic Equivalent of Task (MET)-minute/week, as described in detail elsewhere [60,61,62].

### 4.3. Dietary Record

A validated 143-item semi-quantitative food frequency questionnaire was administered in a face to face interviews by a trained nutritionist to explore dietary intake over the previous 12 months [63]. Furthermore, adherence to MedDiet was assessed by a 17-point score questionnaire, which is a version of the 14-point score performed in the PREDIMED study [56,64,65]. The 17-point score questionnaire includes additional questions to the 14-point score and more restrictive cut-offs for some caloric-dense foods [56,65].

### 4.4. Urine and Plasma Collection, and Biochemical Determinations

The first spot urine was taken in the morning, and blood samples were obtained after 12 h overnight fasting. Biological specimens were stored frozen at a −80 °C according to approved protocols by trained technicians [56]. Biochemical analyses including glucose, hemoglobin A1c (HbA1c), triglyceride, HDL-c, total cholesterol, ALT, and AST were performed on fasting plasma by using specific kits according to manufacturer’s protocols [56]. The insulin was measured using specific ELISA kits in a Triturus autoanalyzer (Grifols, Barcelona, Spain). The Friedewald formula was used to calculate LDL-c and the VLDL-c [66].

### 4.5. Urine Resveratrol Metabolites Measurements

Standards of *trans*-resveratrol-3-*O*-glucuronide, *trans*-resveratrol-4′-*O*-glucuronide, *cis*-resveratrol-3-*O*-glucuronide, *cis*-resveratrol-4′-*O*-glucuronide, and *trans*-resveratrol-3-*O*-sulfate were obtained from Toronto Research Chemicals (Toronto, ON, Canada). The resveratrol metabolites were extracted and quantified using a modified method developed by Feliciano et al. (2016) [67]. The analytical method was validated according to the Food and Drug Administration (FDA) guidelines. Briefly, 600 μL of diluted urine samples (urine:water, 1:10) were thawed on ice and centrifuged at 15,000× *g* for 15 min at 4 °C. Then the supernatant (350 μL) was transferred to a microtube and acidified with 4% phosphoric acid. The mixture (600 μL) was loaded onto Oasis 96-well reversed-phase hydrophilic-lipophilic balanced (HLB) sorbent μ-SPE plates (Waters, Eschborn, Germany) and eluded with 60 μL of methanol after washing. Isotope labelled standards (±)-Catechin-2,3,4-13C3 (0.54 mg/mL, Sigma-Aldrich, Steinheim, Germany) and ferulic acid-1,2,3-13C3 (0.99 mg/mL, Sigma-Aldrich, Steinheim, Germany) were spiked in samples before μ-SPE to indicate the recovery rate. Taxifolin (0.25 mg/mL, Sigma-Aldrich, Steinheim, Germany) were used as internal standard. The identification and quantification of resveratrol metabolites was performed on a Shimadzu Triple Quadrupole Mass Spectrometer (LCMS8060, SHIMADZU, Kyoto, Japan) through an electro-spray interface (ESI) source. Eluded samples (5 μL) were injected through a Raptor Biphenyl column 2.1 × 50 mm, 1.8 µm (Restek, Bellefonte, PA, USA) with a compatible Raptor Biphenyl Guard Cartridges 5 × 2.1 mm (Restek, Bellefonte, PA, USA) in the UPLC system. The mobile phases consisted of solvent A: water (HPLC grade, Sigma-Aldrich, Steinheim, Germany) with 0.1% formic acid (LC-MS grade, Thermo Fisher Scientific, Loughborough, UK), and solvent B: acetonitrile (HPLC grade, Sigma-Aldrich, Steinheim, Germany) with 0.1% formic acid. A fourteen-minute gradient joined by a two minutes equilibration was applied to the run under a flow rate of 0.5 mL/min at 30 °C. The gradient was as follows (t(min), %B): (0, 1), (1, 1), (4, 12), (8, 12) (8.1, 15), (11, 15), (11.5, 30), (12, 99), (14, 99), (14.1, 1), (16, 1). The MS/MS parameters and transitions of the target compounds were obtained in optimization run. The resveratrol metabolites in samples were identified by comparing retention times with standards in corresponding to the multiple reaction monitoring (MRM) transitions and quantified by calibration curves made from standard mixes. One pair of isomers *cis*-resveratrol-3-*O*-glucuronide and *cis*-resveratrol-4′-*O*-glucuronide were quantified together as they appear in the same retention time. The identification of each metabolite was based on retention time of its corresponding pure standard following the same conditions and reference ion ratios based on the MS optimizations. Urinary resveratrol metabolites were normalized for urine creatinine concentrations.

### 4.6. Glucose Homeostasis and Liver Markers Measurements

Glucose homeostasis markers, such as insulin resistance and insulin sensitivity, were calculated using the homeostasis model assessment for insulin resistance (HOMA-IR) [68], as well as the homeostasis model assessment for β-cell function (HOMA-%B) [69], the fasting glucose insulin ratio (FGIR) [70], and the fasting insulin resistance index (FIRI) [70]. Moreover, non-invasive liver markers such as the hepatic steatosis index (HSI) [55,71] and the fatty liver index (FLI) [72] were also determined estimated considering clinical, biochemical and anthropometric data. Formulas followed for all these determinations were as follows:

$$HOMA-IR=Insulin\left(\mathrm{mU}/L\right)*glucose\;\left(\mathrm{mmol}/L\right)/22.5$$

$$HOMA-\%B=\;Insulin\left(\mathrm{mU}/L\right)/glucose\;\left(\mathrm{mmol}/L\right)-3.5$$

$$FGIR=glucose\;\left(\mathrm{mmol}/L\right)/Insulin\left(\mathrm{mU}/L\right)$$

$$FIRI=Insulin\left(\mathrm{mU}/L\right)*glucose\;\left(\mathrm{mmol}/L\right)\;/25$$

$$\mathrm{HSI}=\;8\times \mathrm{ALT}/\mathrm{AST}\;\mathrm{ratio}+\;\mathrm{BMI}\;\left(+2,\;\mathrm{if}\;\mathrm{diabetes};\;+2,\;\mathrm{if}\;\mathrm{female}\right)$$

$$\mathrm{FLI}\;=\;\frac{e^{0.953\times \log\left(\mathrm{triglycerides}\right)+0.139\times \mathrm{BMI}+0.718\times \log\left(\mathrm{GGT}\right)+0.053\times \mathrm{waist}\;\mathrm{circumference}-15.745}}{1+e^{0.953\times \log\left(\mathrm{triglycerides}\right)+0.139\times \mathrm{BMI}+0.718\times \log\left(\mathrm{GGT}\right)+0.053\times \mathrm{waist}\;\mathrm{circumference}-15.745}}\times 100$$

### 4.7. Statistical Analysis

Descriptive statistics were shown as means and standard deviation (SD) for continuous variables, and *n* (%) for categorical variables. A chi-squared test for categorical variables and Student’s *t*-test were used to compare baseline characteristics of participants by sex. Participants were categorized according to tertiles of some urinary resveratrol phase II metabolites excretion (T1 = ≤4.6 nmol/g; T2 = >4.6 to 58.1 nmol/g; T3 = >58.1 to 2481.3 nmol/g creatinine). Unadjusted and adjusted linear regression models were used to analyze the relationship between total urine resveratrol metabolite and cardiometabolic profile and NAFLD risk markers.

The normality of the residuals was tested in order to assess the validity of the regression models. Variables such as triglycerides, Triglyceride/HDL ratio, ALT, AST, and GGT were markedly skewed and were log-transformed. Linear regression analysis was adjusted for sex (except for covariates that include sex), age, smoking status (never, former, current), marital status (single, married, widow, divorced, separated, others), physical activity (MET-min/week), energy intake (kcal/d), and BMI (except for covariates that include BMI). Tests of linear trend were performed assigning the median value of each tertile of total resveratrol urine metabolite and then using it as a continuous variable.

We applied flexible cubic spline models to evaluate the association of total urinary resveratrol metabolites (continuous variable) with liver enzymes above the ULN. For ALT (men ≥ 30 UI/L, women ≥ 19 UI/L) [73], AST (men ≥ 37 UI/L, women ≥ 31 UI/L) [74], and GGT (men ≥ 60 UI/L, women ≥ 40 UI/L) [75]. Models were adjusted by all variables previously mentioned except for sex and included total sleeping hours (h/d). In the cubic spline analysis for total urinary resveratrol metabolites, we used 0 as a reference, with 4 knots (ALT and GGT) and 3 knots (AST). Statistical tests were two-tailed, and the significance level was *p* < 0.05. All statistical analyses were conducted with STATA version 16.0, StataCorp LP, College Station, TX, USA.

## 5. Conclusions

Current data showed that high urinary levels of some resveratrol phase II metabolites were associated with better blood lipid profile and liver enzymes in individuals diagnosed with MetS. Moreover, urinary resveratrol concentration threshold is associated with a reduced risk of higher liver enzymes. These results suggested that some resveratrol metabolites might have associated with benefits on risk factors linked to NAFLD development. Further studies are warranted to elucidate the impact and effectiveness of resveratrol in liver outcomes in individuals with MetS.

## Figures and Tables

**Table 1:** Sociodemographic, clinical and liver characteristics of study participants diagnosed with MetS by sex at baseline.

		Men	Women	*p* ^¶^
	All	(*n* = 153)	(*n* = 113)	
Age (years)	65.8 (5.1)	64.6 (5.4)	67.5 (3.9)	<0.001
BMI (kg/m^2^)	32.2 (3.4)	31.8 (3.1)	32.8 (3.7)	0.019
Waist circumference (cm)	107.3 (9.0)	109.9 (8.5)	103.9 (8.6)	<0.001
VAT (g)	2403.6 (888.9)	2850.3 (826.2)	1831.5 (589.7)	<0.001
SBP (mmHg)	144.8 (16.3)	144.9 (15.8)	144.6 (17.0)	0.887
DBP (mmHg)	87.5 (8.5)	87.9 (8.2)	86.9 (8.9)	0.338
Type 2 diabetes, *n* (%)	100 (37.6)	61 (39.9)	39(34.5)	0.373
Smoking, *n* (%)				<0.001
Never	105 (39.5)	29 (18.9)	76 (67.3)	
Former	124 (46.6)	97 (63.4)	27 (23.9)	
Current	37 (13.9)	27 (17.7)	10 (8.8)	
Lipid-lowering treatment	93 (56.0)	54 (57.5)	39(54.2)	0.673
Any anti-diabetic treatment	69 (25.9)	43 (28.1)	26 (23.0)	0.349
Glucose (mmol/L)	6.7 (1.9)	6.8 (2.1)	6.5 (1.6)	0.286
HbA1c (%)	6.1 (0.9)	6.1 (1.0)	6.1 (0.8)	0.721
Insulin (mU/L)	14.0 (9.0)	13.0 (7.1)	15.6 (11.0)	0.020
HOMA-IR	4.2 (3.2)	3.9 (2.4)	4.7 (4.2)	0.056
Total cholesterol (mg/dL)	200.4 (36.5)	192.2 (34.2)	211.4 (36.9)	<0.001
Triglycerides (mg/dL)	148.3 (61.8)	151.1 (69.0)	144.6 (50.6)	0.402
HDL-c (mg/dL)	45.8 (10.0)	43.0 (8.9)	49.5 (10.1)	<0.001
LDL-c (mg/dL)	125.8 (33.1)	119.8 (31.3)	133.6 (33.9)	<0.001
VLDL-c (mg/dL)	29.7 (12.4)	30.2 (13.8)	28.9 (10.1)	0.402
ALT (U/L)	28.1 (20.6)	31.2 (24.5)	23.8 (12.3)	0.004
AST (U/L)	23.7 (13.4)	25.2 (16.3)	21.8 (7.4)	0.042
GGT (U/L)	42.1 (41.1)	44.9 (41.5)	38.5 (40.3)	0.209
ALT > ULN, *n* (%) *	122 (46.0)	55 (36.0)	67 (59.8)	<0.001
AST> ULN, *n* (%) *	28 (10.5)	12 (7.8)	16 (14.2)	0.097
GGT> ULN, *n* (%) *	51 (19.3)	24 (15.9)	27 (23.9)	0.103
FLI	78.7 (15.1)	80.4 (14.4)	76.4 (15.9)	0.035
HSI	43.0 (4.9)	42.3 (4.7)	44.0 (5.1)	0.004
Physical activity (MET-min/week)	3099.9 (2757.8)	3690 (3101.4)	2301.0 (1954.8)	<0.001

Data were calculated by chi-square or student’s *t*-test as appropriate. Results are expressed as mean (standard deviation). *p* ^¶^ for differences between sexes. Abbreviations: BMI, Body mass index; VAT, visceral adipose tissue; SBP, systolic blood pressure; DBP, diastolic blood pressure; HbA1c, glycated hemoglobin A1c; HOMA-IR, homeostatic model assessment for insulin; HDL-c, high-density lipoprotein cholesterol; LDL-c, Low-density lipoprotein cholesterol; VLDL, very-low-density lipoprotein cholesterol; ALT, alanine aminotransferase; AST, aspartate aminotransferase; GGT, gamma-glutamyl transferase; FLI, fatty liver index; HSI, hepatic steatosis index; MET, metabolic equivalent task. * Upper limit of normal (ULN) range for ALT (men ≥ 30 UI/L, women ≥ 19 UI/L), AST (men ≥ 37 UI/L, women ≥ 31 UI/L), and GGT (men ≥ 60 UI/L, women ≥ 40 UI/L).

**Table 2:** Dietary intake and urine resveratrol metabolites in participants diagnosed with MetS by sex at baseline.

		Men	Women	
	All	(*n* = 153)	(*n* = 114)	*p* ^¶^
Total energy intake (kcal/d)	2608.6 (539.1)	2689.4 (534.5)	2499.1 (528.0)	0.004
Carbohydrate intake (g/d)	282.9 (75.1)	287.0 (73.8)	277.5 (76.9)	0.310
Protein intake (g/d)	102.5 (22.9)	101.3 (23.8)	104.1 (21.8)	0.330
Fat intake (g/d)	108.5 (27.1)	110.9 (26.9)	105.3 (27.2)	0.097
MUFAs (g/d)	55.3 (14.3)	56.4 (14.2)	53.9 (14.5)	0.171
PUFAs (g/d)	18.3 (7.2)	19.1 (7.2)	17.3 (7.1)	0.049
Linoleic (g/d)	15.4 (6.9)	16.1 (6.8)	14.6 (7.0)	0.075
Linolenic (g/d)	1.6 (0.8)	1.6 (0.8)	1.5 (0.8)	0.317
Omega-3 (g/d)	0.9 (0.5)	0.9 (0.5)	0.9 (0.4)	0.305
Fiber (g/d)	30.0 (9.8)	29.3 (10.1)	30.9 (9.3)	0.175
Total cholesterol (g/d)	375.7 (110.8)	382.4 (124.4)	366.5 (88.9)	0.248
Total vegetables (g/d)	333.0 (124.4)	318.2 (123.0)	353.0 (124.0)	0.024
Total fruits (g/d)	423.9 (220.4)	407.3 (221.6)	446.3 (217.8)	0.154
Grapes intake (g/d)	9.7 (21.8)	12.6 (26.4)	5.8 (12.3)	0.012
Cherries and plums (g/d)	14.4 (19.4)	14.9 (19.8)	13.6 (18.9)	0.599
Nuts intake (g/d)	15.1 (18.1)	15.6 (18.1)	14.3 (18.2)	0.566
Homemade fruit juice (mL/d)	4.1 (23.6)	5.6 (29.4)	2.0 (11.5)	0.209
Fruit juice bottle (mL/d)	12.4 (54.8)	12.7 (52.9)	12.1 (57.5)	0.931
Adherence to MedDiet (0–17 points)	8.8 (2.5)	8.8 (2.4)	9.0 (2.5)	0.484
Alcohol consumption (g/d)	12.9 (17.7)	19.7 (19.8)	3.5 (7.2)	<0.001
Total red wine (g/d)	61.4 (105.9)	91.3 (120.8)	20.8 (62.1)	<0.001
Young red wine (g/d)	56.7 (105.3)	84.1 (121.3)	19.6 (61.8)	<0.001
Aged red wine (g/d)	4.7 (25.6)	7.2 (32.5)	1.2 (9.5)	0.056
Rosé wine (g/d)	10.4 (49.8)	16.7 (64.2)	1.9 (11.9)	0.017
Moscatel wine (g/d)	0.5 (7.7)	0.8 (10.1)	0.06 (0.7)	0.429
White wine (g/d)	7.4 (33.6)	10.6 (42.4)	3.1 (14.4)	0.073
*trans*-resveratrol-3-*O*-glucuronide (nmol/g creatinine)	0.7 (1.6)	0.7 (0.9)	0.7 (2.2)	0.973
*trans*-resveratrol-4′-*O*-glucuronide (nmol/g creatinine)	171.9 (375.8)	143.7 (314.7)	210.1 (444.1)	0.154
*trans*-resveratrol-3-*O*-sulfate (nmol/g creatinine)	0.2 (0.5)	0.2 (0.6)	0.1 (0.2)	0.023
*cis*-resveratrol-3-*O*-glucuronide and *cis*-resveratrol-4′-*O*-glucuronide (nmol/g creatinine)	2.0 (5.3)	2.6 (6.0)	1.1 (4.1)	0.023

Data were calculated by student’s *t*-test. Results are expressed as mean (standard deviation). *p* ^¶^ for differences between sexes. Abbreviations: MUFAs, monounsaturated fatty acids; PUFAs, Polyunsaturated fatty acids; MedDiet, Mediterranean diet.

**Table 3:** Linear regression analysis distributed in tertiles evaluating the associations between total urine resveratrol (independent variable) and glucose metabolism markers (outcome) in participants with MetS.

	Total Urine Resveratrol Metabolites (nmol/g Creatinine)			
	T1	T2	T3	*p*-Trend
	(≤4.6)	(>4.6 to 58.1)	(>58.1 to 2481.2)	
*n*	89	89	88	
		β Coefficient (95% IC)	β Coefficient (95% IC)	
Glucose markers				
Glucose (mmol/L)				
Crude model	0 REF.	0.02 (−0.55, 0.60)	0.11 (−0.46, 0.69)	0.677
Adjusted model	0 REF.	0.04 (−0.56, 0.63)	0.04 (−0.55, 0.63)	0.933
HbA1c (%)				
Crude model	0 REF.	−0.07 (−0.35, 0.21)	−0.01 (−0.28, 0.27)	0.842
Adjusted model	0 REF.	−0.07 (−0.36, 0.22)	−0.04 (−0.32, 0.25)	0.982
Insulin sensitivity/resistance markers				
Insulin (mU/L)				
Crude model	0 REF.	−1.23 (−3.94, 1.48)	−1.15 (−3.86, 1.55)	0.623
Adjusted model	0 REF.	−0.22 (−2.87, 2.43)	−0.58 (−3.20, 2.03)	0.672
HOMA-IR				
Crude model	0 REF.	−0.45 (−1.44, 0.54)	−0.46 (−1.44, 0.52)	0.558
Adjusted model	0 REF.	−0.11 (−1.09, 0.87)	−0.31 (−1.28, 0.65)	0.534
HOMA-%B				
Crude model	0 REF.	−15.19 (−37.11, 6.73)	−11.00 (−32.79, 10.79)	0.682
Adjusted model	0 REF.	−11.07 (−32.44, 10.30)	−6.32 (−27.35, 14.71)	0.904
FGIR				
Crude model	0 REF.	−0.12 (−0.30, 0.06)	−0.07 (−0.25, 0.11)	0.859
Adjusted model	0 REF.	−0.14 (−0.33, 0.05)	−0.07 (−0.26, 0.11)	0.922
FIRI				
Crude model	0 REF.	−0.40 (−1.29, 0.49)	−0.42 (−1.30, 0.47)	0.558
Adjusted model	0 REF.	−0.10 (−0.98, 0.78)	−0.28 (−1.14, 0.58)	0.534

Models were adjusted for sex, age, smoking status, marital status, physical activity, energy intake, and BMI. Abbreviations: HbA1c, glycated hemoglobin A1c; HOMA-IR, homeostatic model assessment for insulin; HOMA-%B, HOMA of β-cell function; FGIR, fasting glucose insulin ratio, FIRI, fasting insulin resistance index; REF, reference.

**Table 4:** Linear regression analysis evaluating the associations between total urine resveratrol (independent variable) and blood lipids (outcome) in participants with MetS.

	Total Urine Resveratrol Metabolites (nmol/g Creatinine)			
	T1	T2	T3	*p*-Trend
	(≤4.6)	(>4.6 to 58.1)	(>58.1 to 2481.2)	
*n*	89	89	88	
		β Coefficient (95% IC)	β Coefficient (95% IC)	
Blood lipids				
Total cholesterol (mg/dL)				
Crude model	0 REF.	−13.10 (−23.77, −2.43)	−13.26 (−23.96, −2.56)	0.132
Adjusted model	0 REF.	−8.93 (−19.54, 1.68)	−11.67 (−22.21, −1.13)	0.108
LDL-c (mg/dL)				
Crude model	0 REF.	−11.36 (−21.15, −1.57)	−8.21 (−18.02, 1.61)	0.502
Adjusted model	0 REF.	−9.32 (−19.13, 0.48)	−7.77 (−17.57, 2.02)	0.435
HDL-c (mg/dL)				
Crude model	0 REF.	0.36 (−2.60, 3.32)	−0.38 (−3.36, 2.59)	0.674
Adjusted model	0 REF.	1.41 (−1.51, 4.32)	0.27 (−2.63, 3.18)	0.761
Log triglyceride (mg/dL)				
Crude model	0 REF.	−0.05 (−0.18, 0.07)	−0.14 (−0.26, −0.02)	0.032
Adjusted model	0 REF.	−0.06 (−0.19, 0.07)	−0.15 (−0.28, −0.02)	0.030
VLDL-c (mg/dL)				
Crude model	0 REF.	−1.48 (−5.11, 2.15)	−3.95 (−7.60, −0.30)	0.043
Adjusted model	0 REF.	−1.70 (−5.47, 2.08)	−4.21 (−7.97, −0.46)	0.039
Log triglyceride/HDL ratio				
Crude model	0 REF.	−0.07 (−0.23, 0.09)	−0.14 (−0.30, 0.02)	0.122
Adjusted model	0 REF.	−0.10 (−0.26, 0.07)	−0.16 (−0.33, 0.002)	0.106
Total cholesterol/HDL ratio				
Crude model	0 REF.	−0.38 (−0.69, −0.08)	−0.32 (−0.62, −0.01)	0.304
Adjusted model	0 REF.	−0.39 (−0.70, −0.07)	−0.35 (−0.66, −0.03)	0.241

Models were adjusted for sex, age, smoking status, marital status, physical activity, energy intake, and BMI. Abbreviations: LDL-c, Low density lipoprotein cholesterol; HDL-c, high density lipoprotein cholesterol; VLDL, very low-density lipoprotein cholesterol; Triglyceride/HDL ratio, triglyceride/high density lipoprotein cholesterol ratio; Low density lipoprotein cholesterol/high density lipoprotein cholesterol; total cholesterol/HDL, total cholesterol/high density lipoprotein cholesterol; REF, reference.

**Table 5:** Linear regression analysis evaluating the associations between total urine resveratrol (independent factor) and liver status markers (dependent factor) in participants with MetS.

	Total Urine Resveratrol Metabolites (nmol/g Creatinine)			
	T1	T2	T3	*p*-Trend
	(≤4.6)	(>4.6 to 58.1)	(>58.1 to 2481.2)	
*n*	89	89	88	
		β Coefficient (95% IC)	β Coefficient (95% IC)	
Liver markers				
Log ALT (U/L)				
Crude model	0 REF.	0.03 (−0.12, 0.18)	−0.10 (−0.25, 0.05)	0.074
Adjusted model	0 REF.	0.03 (−0.11, 0.18)	−0.12 (−0.27, 0.02)	0.028
Log AST (U/L)				
Crude model	0 REF.	0.003 (−0.10, 0.10)	−0.09 (−0.19, 0.01)	0.040
Adjusted model	0 REF.	−0.01 (−0.11, 0.09)	−0.12 (−0.22, −0.02)	0.011
Log GGT (U/L)				
Crude model	0 REF.	0.02 (−0.15, 0.20)	−0.23 (−0.41, −0.06)	0.002
Adjusted model	0 REF.	0.01 (−0.17, 0.19)	−0.24 (−0.42, −0.06)	0.002
HSI *				
Crude model	0 REF.	−0.28 (−1.74, 1.18)	−0.59 (−2.45, 1.27)	0.893
Adjusted model	0 REF.	0.14 (−1.35, 1.63)	0.11 (−1.37, 1.59)	0.948
FLI ^¶^				
Crude model	0 REF.	−0.97 (−5.46, 3.52)	−2.55 (−7.07, 1.98)	0.294
Adjusted model	0 REF.	−1.39 (−5.97, 3.18)	−2.54 (−7.10, 2.02)	0.346

Models were adjusted for sex, age, smoking status, marital status, physical activity, energy intake, and BMI. * Adjusted for all variables except for sex and BMI. ^¶^ Adjusted for all variables except for BMI. Abbreviations: ALT, alanine aminotransferase; AST, aspartate aminotransferase; GGT, gamma-glutamyl transferase; HSI, hepatic steatosis index; FLI, fatty liver index; REF, reference.
